# Identification of the molecular regulation of differences in lipid deposition in dedifferentiated preadipocytes from different chicken tissues

**DOI:** 10.1186/s12864-021-07459-8

**Published:** 2021-04-03

**Authors:** Zheng Ma, Na Luo, Lu Liu, Huanxian Cui, Jing Li, Hai Xiang, Huimin Kang, Hua Li, Guiping Zhao

**Affiliations:** 1 School of Life Science and Engineering, Foshan University; Guangdong Provincial Key Laboratory of Animal Molecular Design and Precise Breeding, Foshan, 534861 China; 2 Institute of Animal Sciences, Chinese Academy of Agricultural Sciences; State Key Laboratory of Animal Nutrition, Beijing, 100193 China

**Keywords:** Dedifferentiated preadipocytes, Different tissue derivation, Lipid deposition, DEGs, Chicken

## Abstract

**Background:**

A body distribution with high intramuscular fat and low abdominal fat is the ideal goal for broiler breeding. Preadipocytes with different origins have differences in terms of metabolism and gene expression. The transcriptome analysis performed in this study of intramuscular preadipocytes (DIMFPs) and adipose tissue-derived preadipocytes (DAFPs) aimed to explore the characteristics of lipid deposition in different chicken preadipocytes by dedifferentiation in vitro.

**Results:**

Compared with DAFPs, the total lipid content in DIMFPs was reduced (*P < 0.05*). Moreover, 72 DEGs related to lipid metabolism were screened, which were involved in adipocyte differentiation, fatty acid transport and fatty acid synthesis, lipid stabilization, and lipolysis. Among the 72 DEGs, 19 DEGs were enriched in the PPAR signaling pathway, indicating its main contribution to the regulation of the difference in lipid deposition between DAFPs and DIMFPs. Among these 19 genes, the representative *APOA1*, *ADIPOQ*, *FABP3*, *FABP4*, *FABP7*, *HMGCS2*, *LPL* and *RXRG* genes were downregulated, but the *ACSL1*, *FABP5*, *PCK2*, *PDPK1*, *PPARG*, *SCD*, *SCD5*, and *SLC27A6* genes were upregulated (*P* < 0.05 or *P* < 0.01) in the DIMFPs. In addition, the well-known pathways affecting lipid metabolism (MAPK, TGF-beta and calcium) and the pathways related to cell communication were enriched, which may also contribute to the regulation of lipid deposition. Finally, the regulatory network for the difference in lipid deposition between chicken DAFPs and DIMFPs was proposed based on the above information.

**Conclusions:**

Our data suggested a difference in lipid deposition between DIMFPs and DAFPs of chickens in vitro and proposed a molecular regulatory network for the difference in lipid deposition between chicken DAFPs and DIMFPs. The lipid content was significantly increased in DAFPs by the direct mediation of PPAR signaling pathways. These findings provide new insights into the regulation of tissue-specific fat deposition and the optimization of body fat distribution in broilers.

**Supplementary Information:**

The online version contains supplementary material available at 10.1186/s12864-021-07459-8.

## Background

Fat has unique distribution characteristics and different economic values in various tissues of animals. In broilers, high-intensity artificial breeding has effectively increased the meat yield but has also increased the abdominal fat content and reduced intramuscular fat deposition [1]. Excessive abdominal fat deposition has negative impacts on the feed efficiency and carcass yield [2, 3]. Decreased abdominal fat deposition is beneficial to reduce waste and improve consumer acceptance. In contrast, intramuscular fat is economically desirable in broiler production. Appropriately increased IMF content can improve the meat quality, including color, tenderness, flavor, and juiciness [4–7]. Lowering abdominal fat and increasing intramuscular fat can effectively increase the economic value of broilers.

Previous studies have shown that adipocytes with different origins exhibit differential differentiation capabilities [8]. Compared with subcutaneous preadipocytes, the cell size and lipid droplets in intramuscular adipocytes are smaller [9, 10], and the gene expression and enzyme activation related to lipid metabolism are lower in intramuscular adipocytes [11, 12]. Similarly, abdominal fat-derived preadipocytes exhibited a higher adipogenic differentiation ability than intramuscular fat-derived preadipocytes in chickens [13, 14]. However, it is still unknown whether the difference in the lipogenesis ability of preadipocytes from different tissues will disappear after cultivation in vitro.

In this study, we explored the lipogenesis characteristics of chicken preadipocytes of different origins after cultivation in vitro, including dedifferentiated intramuscular preadipocytes (DIMFPs) and dedifferentiated abdominal preadipocytes (DAFPs). These results will help to understand tissue-specific lipid deposition and optimize body fat distribution in broilers.

## Results

### The difference in lipid deposition in the two types of preadipocytes

**Fig. 1:**
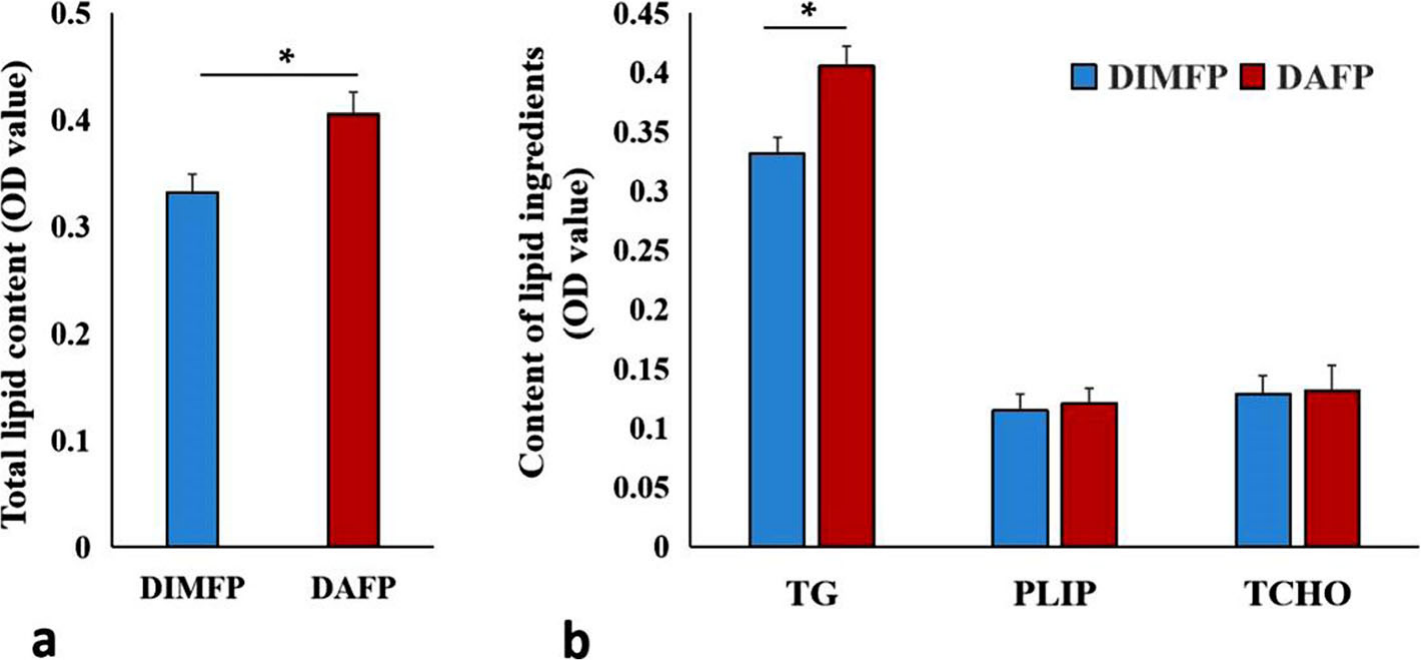
Difference in lipid metabolism between DIMFPs and DAFPs of chickens. **a** and **b** The contents of total lipids and the main ingredients of lipids (TG, PLIP and TCHO). The total lipid and TG contents were increased in the DAFPs compared with the DIMFPs after two days at 100% confluence. Data are presented as the means ± SEM (*n* = 3; * *P* < 0.05)

Collect the DIMFP group and DAFP group cells were collected to detect the total lipid content by an Oil Red O staining assay. As shown in Fig. 1a, the total lipid content in DAFP cells was significantly (*P* < 0.05) higher than that in DIMFP cells. The main ingredients of lipids, triglycerides (TGs), phospholipids (PLIPs), and total cholesterol (TCHO) were also detected. Similarly, the TG content in DAFP cells was significantly (*P* < 0.05) higher than that in DIMFP cells. However, the contents of PLIP and TCHO showed no difference in the two types of preadipocytes (Fig. 1b).

### Identification of DEGs

**Fig. 2:**
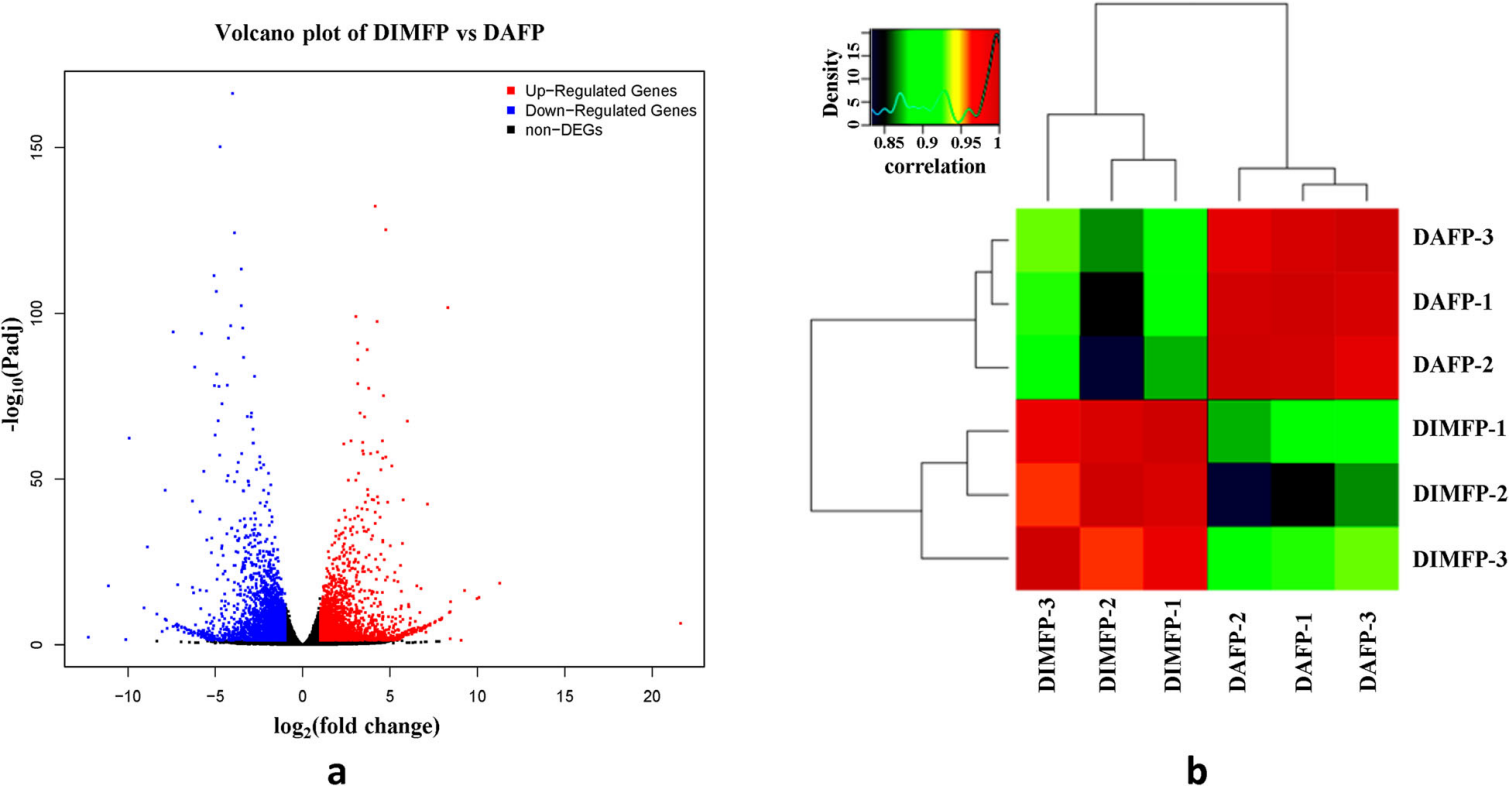
Volcano plot and cluster analysis of differentially expressed genes (DEGs). **a** Volcano plot. Red dots (UP) represent significantly upregulated genes (log_2_FC ≥ 1.0, FDR < 0.05); blue dots (DOWN) represent significantly downregulated genes (log_2_ FC ≤ − 1.0, FDR < 0.05); and black dots (NO) represent DEGs below the level of significance; (**b**) based on 3486 known DEGs in DIMFPs and DAFPs of chickens, cluster analysis was performed. The results show that the gene expression profiling data in the same group were closely related

Total RNA of each of the three cell repetitions of the DIMFP and DAFP groups was extracted for RNA sequencing. A total of 21,469 expressed genes were found in DIMFPs and DAFPs (Additional file 1: Table S1). Using gene expression profiling and comparing the DAFP group with the DIMFP group (DIMFP vs DAFP), a total of 3629 known DEGs (|log_2_ FC| ≥1, with *P < 0.05*) were screened (Fig. 2a), of which 2579 DEGs were downregulated and 907 DEGs were upregulated (Additional file 2: Table S2). Next, cluster analysis was performed on these 21,469 genes, and two results showed the same situation: three cell samples of the same groups were clustered together (Fig. 2b).

### Analysis of the enriched GO terms and pathways in the two types of preadipocytes

**Fig. 3:**
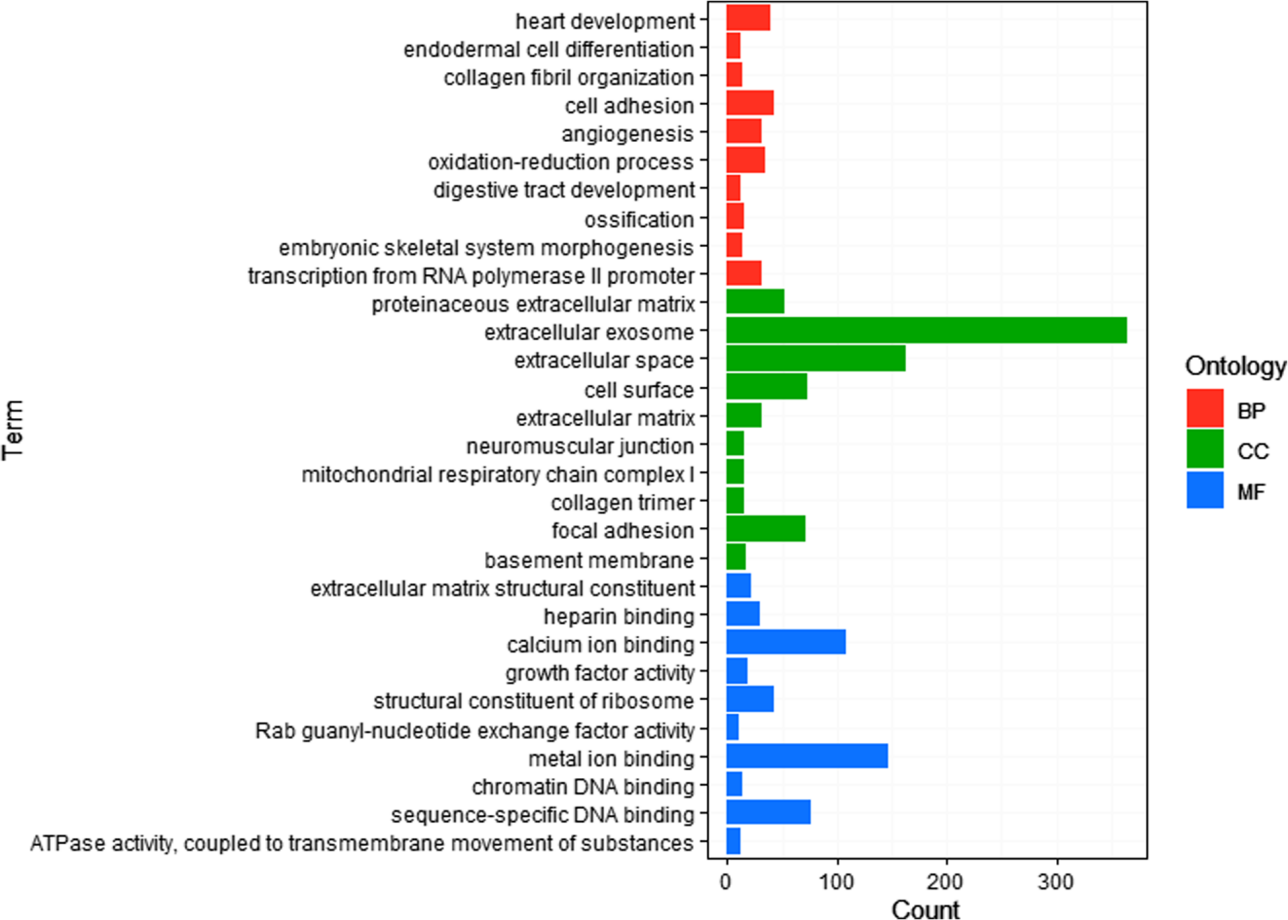
List of enriched Gene Ontology (GO) terms with the top 10. The enriched Gene Ontology (GO) terms were enriched (*P* < 0.05) based on the 3486 DEGs, and the GO terms with the top 10 biological process (BP), cellular component (CC) and molecular function (MF) terms are listed

Based on 3629 known DEGs, Gene Ontology (GO) analysis was performed, and 56 GO terms were enriched (*P* < 0.05), mainly including the following processes: cell adhesion, tight adhesion, cell differentiation, extracellular matrix, DNA binding, calcium ion binding, etc. (Additional file 3: Table S3). The top 10 terms of each of the biological process (BP), cellular component (CC) and molecular function (MF) terms are shown in Fig. 3.

**Fig. 4:**
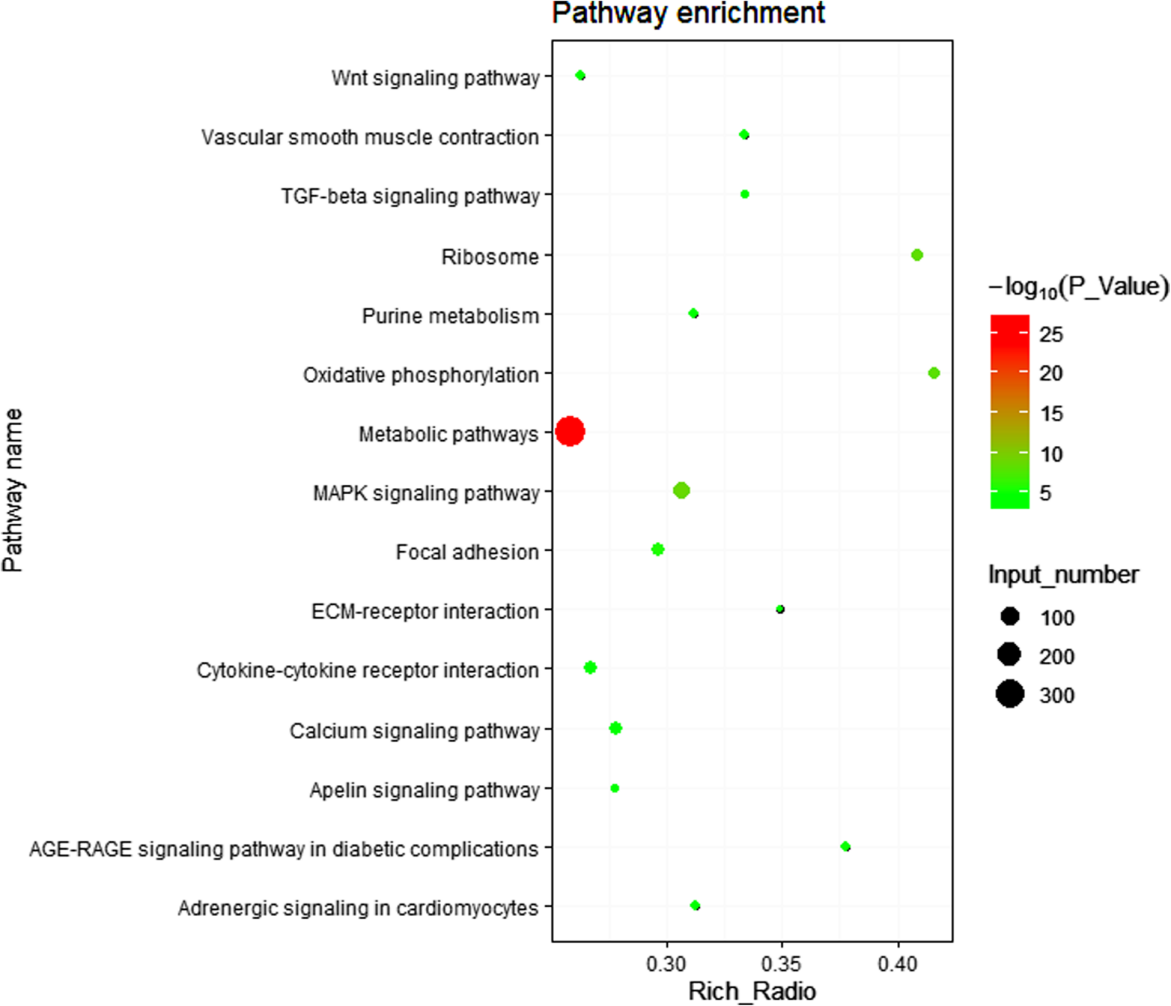
List of enriched pathways with the top 15 based on the 3486 DEGs. The KEGG (Kyoto Encyclopedia of Genes and Genomes) pathway analysis showed that well-known pathways (MAPK, TGF-beta, Wnt, calcium, and PPAR signaling pathways) of lipid metabolism were enriched, and the enriched pathways with the top 15 were screened (adjusted *P* < 0.05)

Meanwhile, 47 pathways were found to be significantly enriched (corrected *P*-value < 0.05) (Additional file 4: Table S4), including some well-known pathways affecting lipid metabolism (PPAR, MAPK, TGF-beta, Wnt, and calcium signaling pathways) and other pathways related to cell communication (focal adhesion, cytokine-cytokine receptor interaction, ECM-receptor interaction, tight junction, regulation of the actin cytoskeleton, cell adhesion molecules, and adherens junction pathways). The top 15 enriched pathways are shown in Fig. 4.

### DEGs related to lipid metabolism in the two types of preadipocytes

**Fig. 5:**
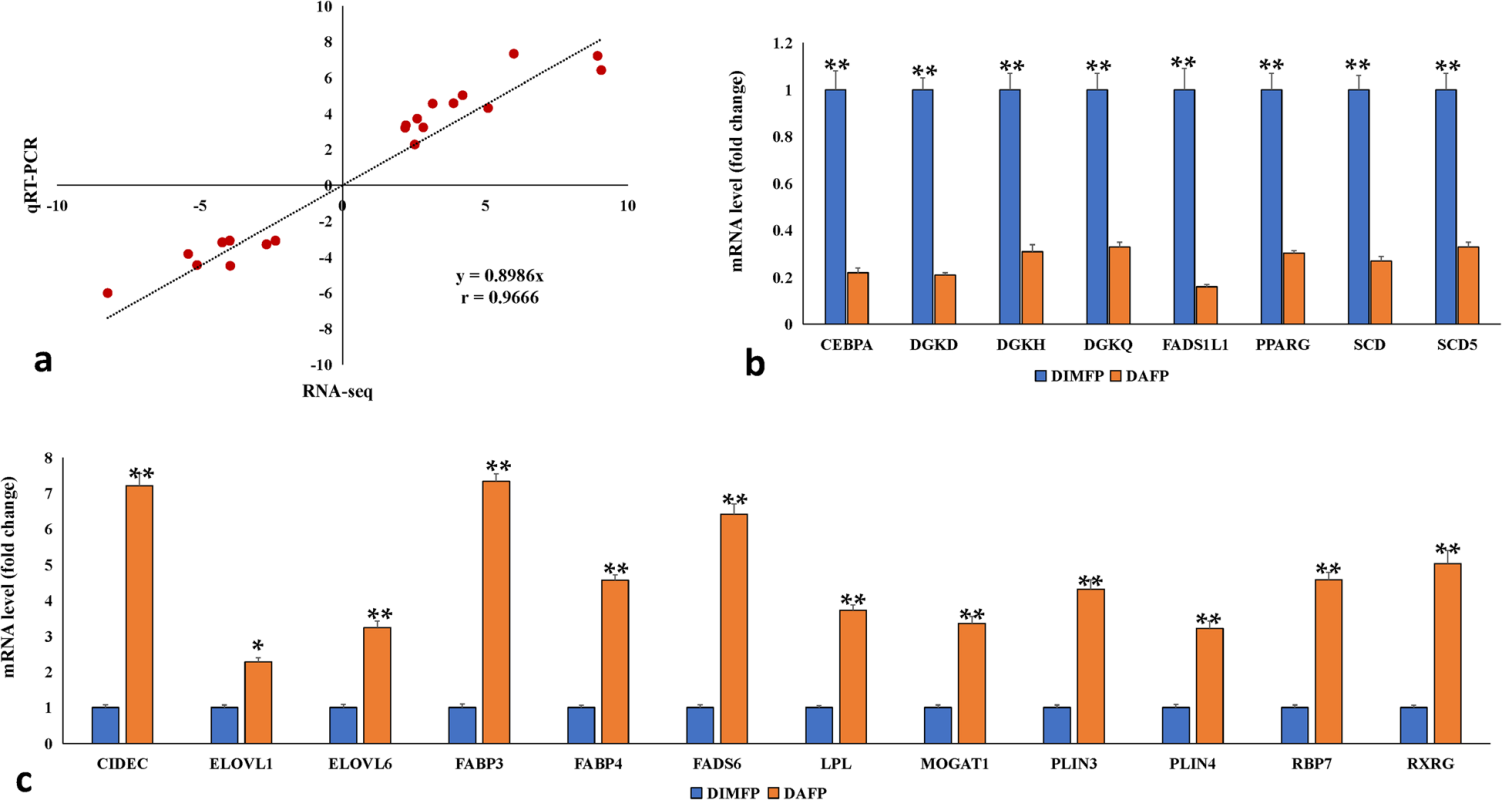
Validation of DEGs related to lipid metabolism between DIMFPs and DAFPs of chickens. **a** Correlation analysis of gene expression profiling and real-time quantitative polymerase chain reaction (qRT-PCR) results by Spearman rank correlation in DIMFPs and DAFPs. A high correlation coefficient (*r* = 0.9666, *P* < 0.05) was detected, which indicates that the gene expression profiling data are reliable. *n* = 20; (**b**) and (**c**) qRT-PCR verification of DEGs detected by gene expression profiling. The expression levels of DEGs related to lipid metabolism determined by qRT-PCR in the DIMFPs and DAFPs. Each of these DEGs was upregulated or downregulated significantly (*P* < 0.05) in DIMFPs and DAFPs. Data are presented as the means ± SEM (*n* = 3; * *P* < 0.05, ** *P* < 0.01)

GO enrichment analysis indicated 72 DEGs related to lipid metabolism, and some representative DEGs were screened (Additional file 5: Table S5). The DEGs related to lipid metabolism were mainly involved in adipocyte differentiation (such as *CEBPA*, *PPARG*, *RBP7,* and *RXRG*), fatty acid transport and fatty acid synthesis (such as *ELOVL1*, *ELOVL6*, *FABP3*, *FABP4*, *FADS6*, *FADS1 L1*, *SCD,* and *SCD5*), lipid stabilization (such as *CIDEC*, *PLIN3*, *PLIN4,* and *MOGAT1*), and lipolysis (such as *DGKD*, *DGKH*, *DGKQ,* and *LPL*). The 20 representative DEGs related to lipid metabolism were randomly selected to validate the gene expression profiling results by qRT-PCR, and the correlation of gene expression profiling and qRT-PCR was analyzed by Spearman rank correlation to confirm the accuracy of the data. The results showed that the fold change in gene expression between the two methods was significantly correlated (Fig. 5a) (*r* = 0.9666, *P* < 0.01).

Among these 20 verified genes, the expression levels of the *CEBPA*, *DGKH*, *DGKQ*, *DGKD*, *FADS1L1*, *SCD*, *SCD5,* and *PPARG* genes were significantly (*P* < 0.05 or *P* < 0.01) downregulated in DAFPs compared to DIMFPs (Fig. 5b). However, the expression levels of the *CIDEC*, *ELOVL1*, *ELOVL6*, *FABP3*, *FABP4*, *FADS6*, *LPL*, *MOGAT1*, *PLIN3*, *PLIN4*, *RBP7,* and *RXRG* genes were significantly (all *P* < 0.01) upregulated in DAFPs compared to DIMFPs (Fig. 5c).

### Pathways involved in lipid metabolism

It was found that 19 genes related to lipid metabolism enriched in the PPAR signaling pathway (Additional file 6: Fig. S1)*.* Among these 19 genes, the data from RNA-seq showed that *APOA1*, *ADIPOQ*, *FABP3*, *FABP4*, *FABP7*, *HMGCS2*, *LPL* and *RXRG* genes were down-regulated, but *ACSL1*, *FABP5*, *PCK2*, *PDPK1*, *PPARG*, *SCD*, *SCD5*, *SLC27A6* genes were up-regulated (*P* < 0.05 or *P* < 0.01) in the DIMFPs. (Additional file 2: Table S2).

**Fig. 6:**
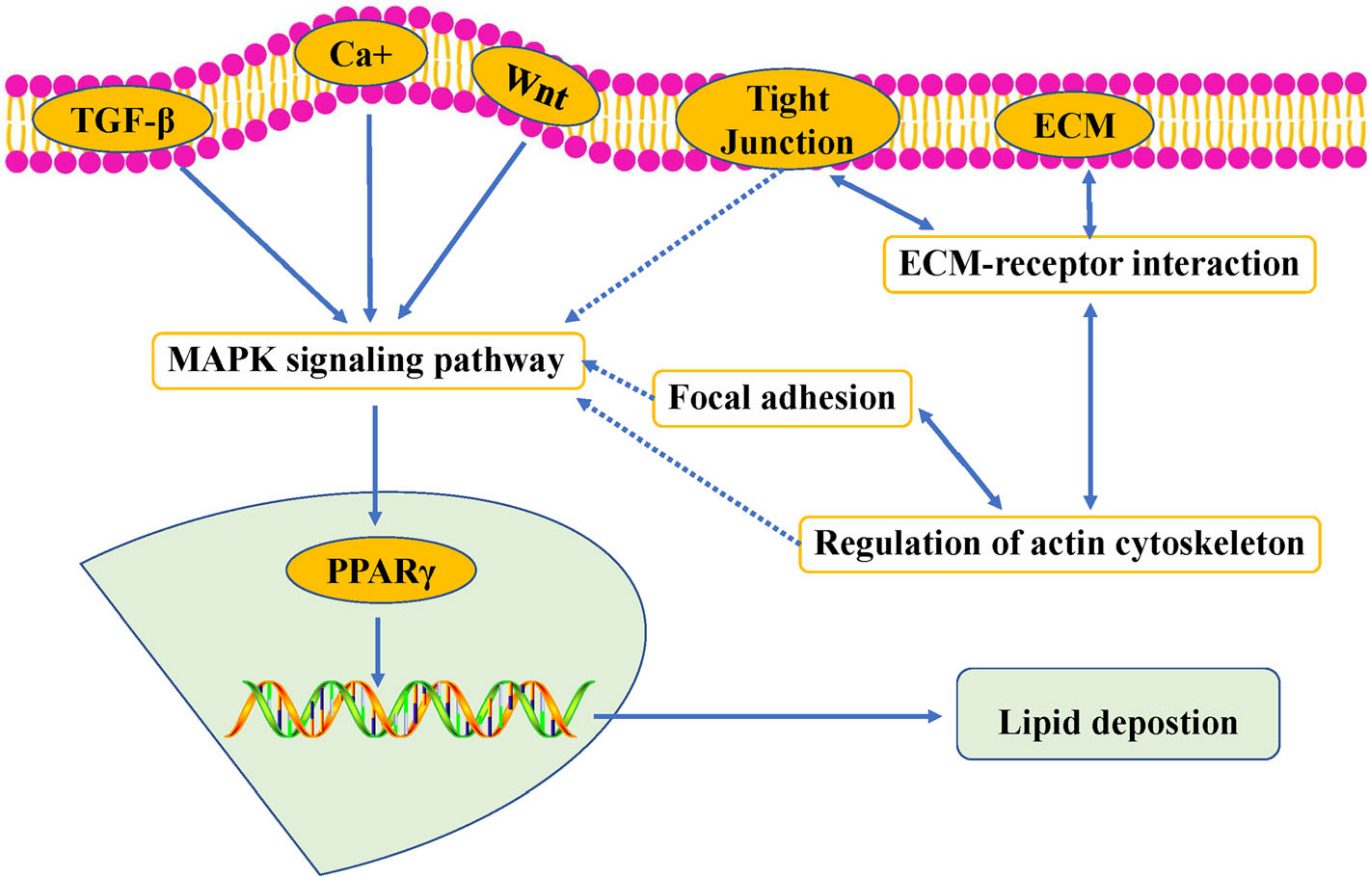
Proposed regulatory network for the difference in lipid deposition in DIMFPs and DAFPs based on DEGs and enriched signaling pathways

Also, there are a large number of DEGs that were enriched in MAPK- (80 genes), Calcium- (50 genes), and TGF beta (30 genes) signaling pathway, which involved in mediating the biology function of lipid metabolism (Additional file 7: Fig. S2, Additional file 8: Fig. S3, and Additional file 9: Fig. S4). Besides, 245 DEGs also were enriched the pathways related to cell communications (Focal adhesion, Cytokine-cytokine receptor interaction, ECM-receptor interaction, Tight junction, Regulation of actin cytoskeleton, cell adhesion molecules, Adherens junction). However, it was found that the enriched Wnt signaling pathway, as a well-known pathway affecting lipid metabolism, did not medicate the regulation of lipid metabolism. Based on the above information, we proposed the regulatory network for the difference of lipid deposition between chicken DAFPs and DIMFPs (Fig. 6).

## Discussion

Fat has unique distribution characteristics and different economic values in various tissues of animals. In broilers, intramuscular fat is economically desirable in production. Appropriately increased IMF content can improve meat quality, including tenderness, flavor, and juiciness [4–6]. However, excessive abdominal fat deposition has negative impacts on the feed efficiency and carcass yield [2, 3], and decreased abdominal fat deposition is beneficial to reduce waste production and improve consumer acceptance. Lowering abdominal fat and increasing intramuscular fat can effectively increase the economic value of broilers. Therefore, changing the constitution distribution is an important scientific problem for broilers.

Unlike the marbling distribution of IMF in domestic animals, the IMF of chickens cannot be obtained directly from anatomy. Moreover, chicken muscle tissue has a variety of cell compositions [15], and IMF preadipocytes cannot be separated by physical methods due to their similar density to muscle cells. Therefore, high-purity preadipocytes of IMF can only be obtained by the dedifferentiation of mature adipocytes in vitro as described previously [16]. In this study, abdominal fat preadipocytes and intramural preadipocytes were obtained from mature adipocytes of the same chicken to compare their lipogenesis ability under consistent experimental conditions in vitro, establishing a theoretical foundation for the body fat distribution of chickens and providing ideas and development directions for chicken production.

Adipocytes in different tissues are regulated by the adjacent microenvironment to perform the corresponding physiological function [17, 18]. To eliminate the effects of factors in vivo and in vitro, second-generation cells were used. After the cells were overgrown for 2 days, the lipogenesis of adipocytes was detected, which was different from the usual practice of inducing adipocyte differentiation in vitro, avoiding the possibility that the inducers could conceal the lipogenesis of the cells themselves. The results showed that the lipogenesis of preadipocytes derived from abdominal adipocytes was significantly increased compared to that of preadipocytes derived from muscle tissue, consistent with previous in vivo results [19, 20], and the increase in the TG content was responsible for the improvement in total lipids.

To identify the regulatory mechanism of lipid deposition for the difference between DIMFPs and DAFPs, RNA sequencing was performed to screen the functional genes and important pathways related to lipid deposition, and quality control by cluster analysis and qRT-PCR indicated the reliability of the RNA sequencing data. Based on the 3629 screened DEGs, GO terms and KEGG analysis were performed. Forty-seven enriched pathways were screened, including the well-known pathways affecting lipid metabolism (MAPK, TGF beta, Wnt, calcium, and PPAR signaling pathways as well as the pathways related to cell communication). Furthermore, we identified the DEGs related to lipid metabolism according to the enriched GO terms and signaling pathways. Among the 72 DEGs related to lipid metabolism, 19 genes were enriched in the PPAR signaling pathway with the classic mediation of lipid metabolism [21, 22]. Among these 19 genes, the RNA-seq data showed that the *APOA1*, *ADIPOQ*, *FABP3*, *FABP4*, *FABP7*, *HMGCS2*, *LPL* and *RXRG* genes were downregulated but the *ACSL1*, *FABP5*, *PCK2*, *PDPK1*, *PPARG*, *SCD*, *SCD5*, and *SLC27A6* genes were upregulated (*P* < 0.05 or *P* < 0.01) in the DIMFPs, which had an important regulatory effect on lipid metabolism [14, 21–35]. Therefore, it was considered that these genes and the PPAR signaling pathway had important effects in vitro on regulating the difference in lipid deposition between the DIMFPs and DAFPs of chickens.

It was reported that the MAPK, calcium, and TGF beta signaling pathways interact with the PPAR pathway to regulate lipid metabolism in the lipogenesis process, and a large number of genes were enriched in the MAPK, calcium, and TGF beta signaling pathways [36–38]. Coincidentally, there were a large number of DEGs that were enriched in the MAPK (80 DEGs), calcium (50 DEGs), and TGF-beta (30 DEGs) signaling pathways, which are involved in mediating the biological function of lipid metabolism. According to the enrichment information of these three signaling pathways in this study, the evidence indicated that these three pathways could mediate the biological function of cell differentiation or metabolism. Then, it was deduced that the MAPK, calcium, and TGF beta signaling pathways were also involved in the regulation of lipogenesis between DAFPs and DIMFPs. As in our previous report [39], these pathways related to cell communication also participate in the regulation of lipid deposition through the MAPK signaling pathway in chickens. In this study, multiple enriched pathways related to cell communication (245 DEGs) were screened, including focal adhesion, cytokine-cytokine receptor interaction, regulation of the actin cytoskeleton, tight junction, ECM-receptor interaction, and cell adhesion molecules (CAMs), suggesting that the pathways related to cell communication affected the difference in lipid deposition between DIMFPs and DAFPs of chickens. Based on the above information, it was found that the Wnt signaling pathway, a well-known pathway related to lipid metabolism, does not directly regulate lipid metabolism.

It is well known that the fat content in adipose tissue in chickens is far greater than that in intramuscular fat, which may be due to the higher expression of some genes involved in fat synthesis in DAFP than in DIMFP. For example, in our study, the expression of star genes in fat synthetic pathways, such as *ELOVL1*, *ELOVL6*, *FABP3*, *FABP4*, *MOGAT1*, *PLIN3*, and *PIN4*, which are related to fat synthesis, was significantly increased in DAFP, and the amount of fat synthesized in DAFP was also higher than that in DIMFP, which may be due to the differences in the expression of these genes. Therefore, we speculate that these genes can also be used as biomarkers of fat content. Similarly, these genes may also be used as biomarkers of fat accumulation in chickens, but this requires further experimental verification.

## Conclusions

In brief, our data suggest a difference in lipid deposition between the DIMFPs and DAFPs of chickens in vitro and propose a molecular regulatory network for the difference in lipid deposition between chicken DAFPs and DIMFPs. The lipid content was significantly increased in DAFPs by the direct mediation of PPAR signaling pathways. These findings establish the groundwork and provide new insights into the regulation of tissue-specific fat deposition and optimizing body fat distribution in broilers. In the future, additional studies will be required to complement the effects of these important genes on lipid deposition and pathways in DIMFPs and DAFPs.

## Methods

### Animals and ethics statement

Three BJY chickens were obtained from the Institute of Animal Sciences, CAAS (Beijing, China), which were raised under the same recommended environmental and nutritional conditions. Animal experiments were approved by the Science Research Department, Chinese Academy of Agricultural Sciences (CAAS) (Beijing, China). Three birds were individually euthanized by carbon dioxide anesthesia and exsanguination by severing the carotid artery at 10 days of age, and the pectoralis major and abdominal fat tissues were excised for cell isolation.

### Preadipocyte acquisition

Mature adipocytes from the pectoralis major and abdominal fat tissue were isolated as previously described, and then, preadipocytes were obtained with dedifferentiation treatment as previously described [16]. The abdominal fat tissue and pectoralis major of three chickens were collected and then washed with phosphate-buffered saline (PBS) containing 1% penicillin-streptomycin (Gibco, Thermo Fisher Scientific Inc., Suzhou, China). The abdominal fat tissue and pectoralis major from the same chicken were recorded for the one-to-one correspondence of cell samples in subsequent experiments. After the removal of the blood vessels and connective tissue, the samples were finely minced to 1 mm^3^ with scissors and then digested in DMEM/F12 (1:1) medium (Gibco, Thermo Fisher Scientific Inc., Suzhou, China) containing 0.1% Type I collagenase (Sigma-Aldrich, Shanghai, China) in a water bath with continuous shaking at 37 °C for 60 min. After the termination of digestion and filtration, the cell suspension was centrifuged at 600×*g* for 15 min. The top layer containing mature adipocytes was collected and placed in a 25 cm^2^ cell culture flask, which was inverted and filled with DMEM/F12 (1:1) medium containing 10% FBS. The floating mature adipocytes adhered to the bottom of the flask and were incubated in a 37 °C incubator with 5% CO_2_. After 3 days, the mature adipocytes would gradually converse to preadipocytes by releasing lipids by exocytosis, the medium was replaced, and the flask was reinverted so that the preadipocytes were on the bottom to proliferate massively. After up to 15 days, the confluence of preadipocytes reached 80–90%, and the cells were subcultured.

### Preadipocyte culture and treatment

The medium was changed every 3 days. After the cells reached 80% confluence, the cells were passaged, and second passage (P2) preadipocytes were used for further experiments by the dedifferentiation of mature adipocytes from the pectoralis major (DIMFPs) and abdominal fat tissue (DAFPs). Both DIMFPs and DAFPs were plated in 24-well or 100-mm dishes, and cells were collected after 2 days at 100% confluence for further RNA extraction and measurement of biochemical indices (in 100-mm dishes) or Oil Red O staining assays (in 24-well plates).

### Oil red O staining assay

The cellular lipid contents of DIMFPs and DAFPs in 24-well plates were determined by Oil Red O staining. The staining steps were as follows: discard the cell culture medium, wash the adherent cells 3 times with PBS, fix the cells with 4% formalin for 30 min, wash the cells 3 times with PBS, and use Oil Red O (Wuhan AmyJet Scientific Inc., Wuhan, China) dyeing for 60 min. Subsequently, Oil Red O was discarded, and the cells were washed with PBS 3 times. After the water evaporated, isopropanol was added to extract Oil Red O for 10 min, and then, the absorbance of the solution was measured at 510 nm with a Multimode Microplate Reader (Thermo Fisher Scientific Inc., Suzhou, China).

### Measurement of biochemical indices

The two groups of cell samples were homogenized with absolute ethanol at room temperature for 20 min and centrifuged at 1000×*g*. Then, the supernatant was collected, and a TG content detection kit (Nanjing Jiancheng Bioengineering Institute, Nanjing, China), TCHO content detection kit (Nanjing Jiancheng Bioengineering Institute, Nanjing, China), and PLIP content detection kit (Beijing Lindemann Biochemical Co., Ltd., Beijing, China) were used to detect the TG, TCHO, and PLIP contents in the two groups of cells. The specific measurement steps were performed according to the manufacturer’s instructions.

### RNA extraction and identification

TRIzol reagent (Invitrogen, Carlsbad, CA, USA) was used to extract total RNA from DIMFP cells and DAFP cells, which were cultured in a 100 mm cell culture dish according to the manufacturer’s instructions. The quality of RNA was detected by 1.5% gel electrophoresis, and the concentration of RNA was determined using a Nanodrop2000 spectrophotometer (Thermo Fisher Scientific Inc., Suzhou, China). The OD260/280 value of all cell samples must be in the range of 1.8 to 2.0 before they can be used. The RNA sample was then used for gene expression analysis.

### Gene expression profiling

High-throughput sequencing (HiSeq2500; Illumina, San Diego, California, USA) was performed on RNA samples of two groups of cells, and the raw data were converted into FASTQ files using bcl2fastq. The clean reads were generated by removing reads with adapter and low-quality sequences and mapped to the reference chicken genome and genes (*Gallus gallus*, Galgal6; available at https://www.ncbi.nlm.nih.gov/assembly/GCF_000002315.6) using TopHat 1.3.2 (https://ccb.jhu.edu/software/tophat). Gene expression levels were calculated using the RPKM method, as described by Mortazavi et al. [40]. The edgeR R software package was used to analyze the differentially expressed genes (DEGs) between the DIMFP group and the DAFP group. The DEG screening conditions were | log_2_ FC | ≥1.0 and FDR < 0.05. Cluster analysis of DEGs was performed by the pheatmap software package of R software.

### Gene ontology and Kyoto encyclopedia of genes and genomes analysis

The ClueGO plug-in and CluePedia plug-in in Cytoscape (https://cytoscape.org/) software were used to perform Gene Ontology (GO) enrichment analysis and functional classification of the DEGs. The significance level of GO term enrichment was set at *P < 0.05*. At the same time, the Kyoto Encyclopedia of Genes and Genomes (KEGG) [41] database was used for pathway enrichment analysis of DEGs. *P* < 0.05 was considered to be indicative of statistical significance. According to the results of GO enrichment analysis and KEGG pathway function enrichment, DEGs related to abdominal fat tissue metabolism were screened.

### Real-time quantitative polymerase chain reaction

A Tiangen® FastQuant RT kit (Tiangen, Beijing, China) was used for the reverse transcription of the RNA samples of the two groups of cells according to the steps provided by the manufacturer’s instructions, and Primer 5.0 software was used to design specific primers for each gene according to the GenBank sequence (Additional file 10: Table S6). The ABI 7500 real-time PCR detection system (Applied Biosystems, CA, USA) was used for relative quantitative analysis of gene expression in different samples. The total reaction system was 20 μL and contained 10 μL of SYBR Green mix (TaKaRa, Shanghai, China), 0.5 μL of forward primers, and 0.5 μL of reverse primers at a concentration of 10 nmol, 1 μL of cDNA, and 8 μL of ddH_2_O. The reaction program was denaturation at 95 °C for 30 s, followed by 40 cycles of amplification at 95 °C for 5 s and 60 °C for 32 s. The 2^-△△Ct^ method [42] was used to calculate the fold change of gene expression with the expression of actin beta (β-actin), which was a reference gene.

### Statistical analysis

Three comparison replicates (DIMFPs vs DAFPs) of the cell experiment were set according to the one-to-one correspondence of cell samples from the abdominal fat tissue and pectoralis major of the same chicken. All experiments were repeated three times, and the data obtained are expressed as the mean ± SEM. Statistically significant differences between the two culture conditions were tested by independent-samples t-tests using SAS 9.2 software (SAS Institute Inc., NC, USA). *P* < 0.05 (*) or *P* < 0.01 (**) was considered to be significant. All figures were constructed using GraphPad Prism version 5.02 (GraphPad Software Inc., CA, USA).

## Supplementary Information

**Additional file 1: Table S1**: Expression of genes (DIMFPs vs DAFPs).

**Additional file 2: Table S2**: Screened known DEGs (DIMFPs vs DAFPs).

**Additional file 3: Table S3**: Enriched GO terms based on DEGs (DIMFPs vs DAFPs).

**Additional file 4: Table S4**: Enriched pathways based on DEGs (DIMFPs vs DAFPs).

**Additional file 5: Table S5**: Screened DEGs related to lipid metabolism (DIMFPs vs DAFPs).

**Additional file 6: Fig. S1**: DEGs involved in the PPAR signaling pathway, which were determined based on the 03320 pathway map in the KEGG database. The red box plot shows downregulated genes, and the green box plot shows upregulated genes in the pathway (DIMFPs vs DAFPs). The figure has obtained KEGG copyright permission.

**Additional file 7: Fig. S2**: DEGs involved in the MAPK signaling pathway, which were determined based on the 04010 pathway map in the KEGG database. The red box plot shows downregulated genes, and the green box plot shows upregulated genes in the pathway (DIMFPs vs DAFPs). The figure has obtained KEGG copyright permission.

**Additional file 8: Fig. S3**: DEGs involved in the calcium signaling pathway, which were determined based on the 04020 pathway map in the KEGG database. The red box plot shows downregulated genes, and the green box plot shows upregulated genes in the pathway (DIMFPs vs DAFPs). The figure has obtained KEGG copyright permission.

**Additional file 9: Fig. S4**: DEGs involved in the TGF beta signaling pathway, which were determined based on the 04350 pathway map in the KEGG database. The red box plot shows downregulated genes, and the green box plot shows upregulated genes in the pathway (DIMFPs vs DAFPs). The figure has obtained KEGG copyright permission.

**Additional file 10: Table S6**: Information on the specific primers used for qRT-PCR.

## Data Availability

All of raw data generated during the study are uploaded to the NCBI-SRA database (https://www.ncbi.nlm.nih.gov/sra/) under the accession number SRR13665175, SRR13665173, SRR13665172, SRR13665171, SRR13665170, SRR13665174.

## Abbreviations

ACSL1: Acyl-CoA synthetase long-chain family member 1
APOA1: Apolipoprotein A1
ADIPOQ: Adiponectin, C1Q and collagen domain containing
BP: Biological process
CC: Cellular component
CEBP: CCAAT enhancer binding protein
CIDEC: Cell death inducing DFFA like effector c
DEG: Differentially expressed genes
DIMFPs: Dedifferentiated intramuscular preadipocytes
DAFPs: Dedifferentiated abdominal preadipocytes
DGK: Diacylglycerol kinase
ELOVL: Elongase of very long-chain fatty acids-like
FABP: Fatty acid binding protein
FADS: Fatty acid desaturase
HMGCS2: 3-Hydroxy-3-methylglutaryl-coenzyme a synthase 2
IMF: Intramuscular fat
GO: Gene ontology
KEGG: Kyoto encyclopedia of genes and genomes
LPL: Lipoprotein lipase
MAPK: Mitogen- activated protein kinase
MF: Molecular function
MOGAT: Monoacylglycerol O-acyltransferase.
PBS: Phosphate-buffered saline
PCK2: Phosphoenolpyruvate Carboxykinase 2
PDPK1: 3-Phosphoinositide dependent protein kinase-1
PLIN: Perilipin
PPAR: Peroxisome proliferators-activated receptors
qRT-PCR: Quantitative real-time polymerase chain reaction
RBP: Retinol binding protein
RXR: Retinoid X receptor
SCD: Stearoyl-CoA desaturase
SLC27A6: Solute carrier family 27, member 6
Wnt: Wingless/Int
