# Supplementary figures and images for: Identification of the molecular regulation of differences in lipid deposition in dedifferentiated preadipocytes from different chicken tissues
Source: BMC Genomics. 2021 Apr 3;22:232. doi: 10.1186/s12864-021-07459-8 (PMC8019497; doi:10.1186/s12864-021-07459-8)

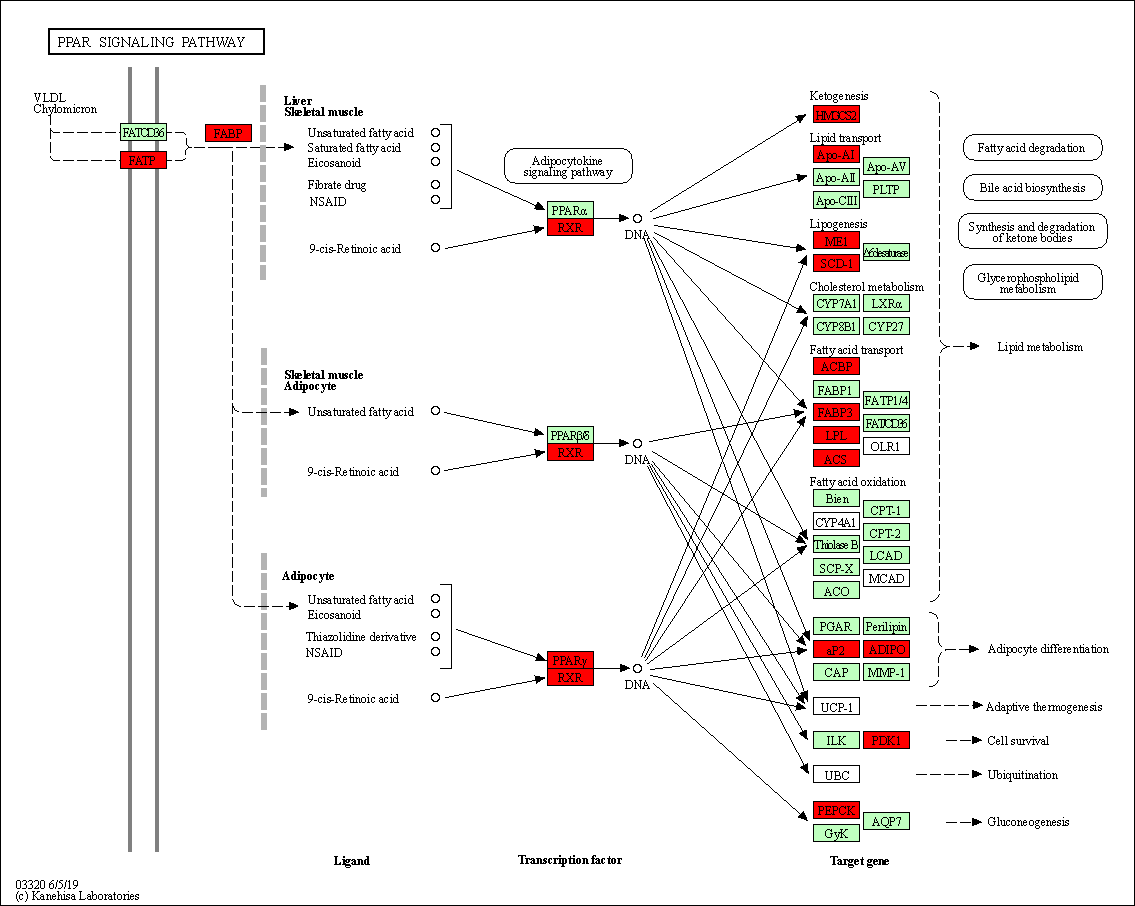

Supplement: Supplementary file 6 — Additional file 6: Fig. S1: DEGs involved in the PPAR signaling pathway, which were determined based on the 03320 pathway map in the KEGG database. The red box plot shows downregulated genes, and the green box plot shows upregulated genes in the pathway (DIMFPs vs DAFPs). The figure has obtained KEGG copyright permission. [file 12864_2021_7459_MOESM6_ESM.png]

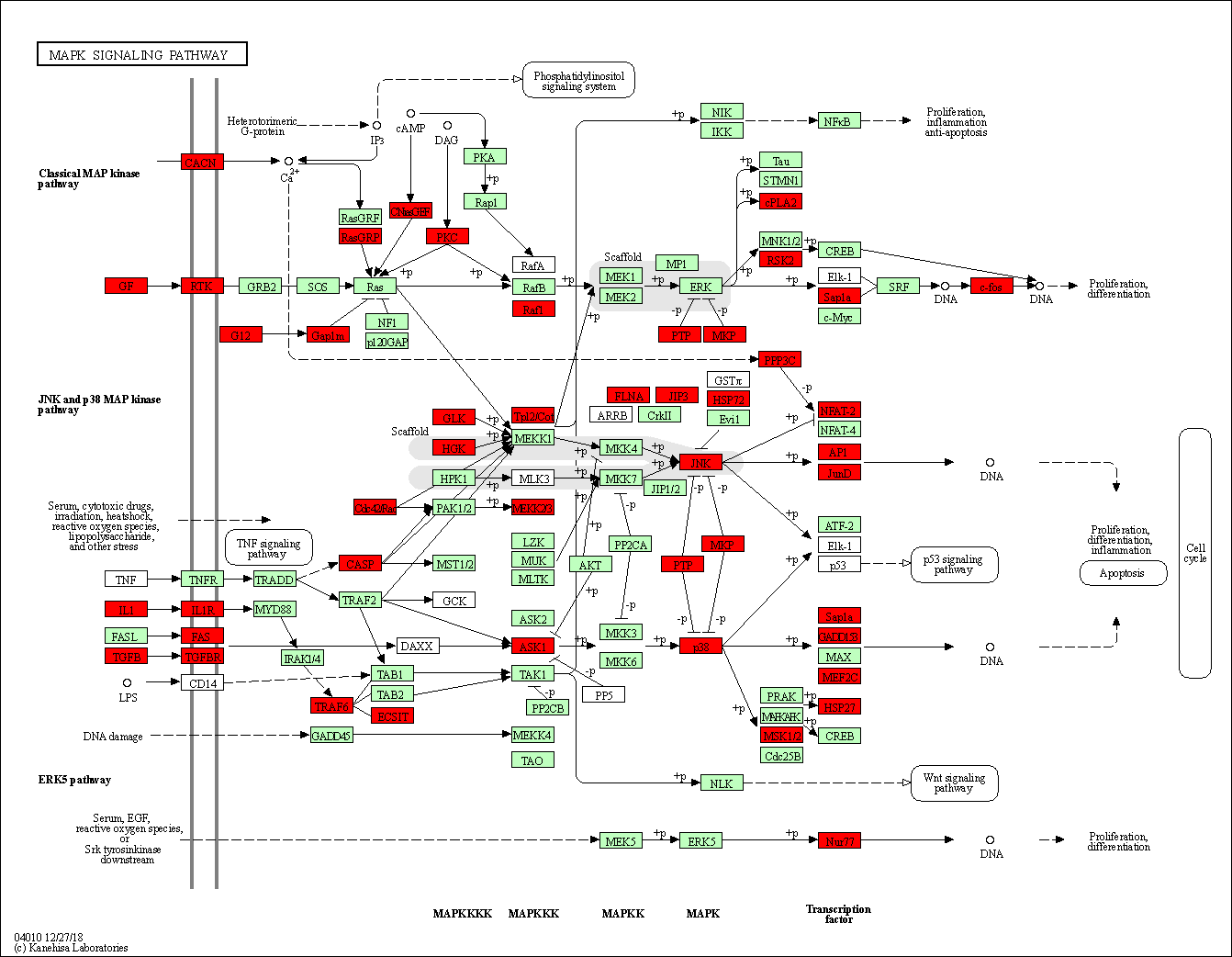

Supplement: Supplementary file 7 — Additional file 7: Fig. S2: DEGs involved in the MAPK signaling pathway, which were determined based on the 04010 pathway map in the KEGG database. The red box plot shows downregulated genes, and the green box plot shows upregulated genes in the pathway (DIMFPs vs DAFPs). The figure has obtained KEGG copyright permission. [file 12864_2021_7459_MOESM7_ESM.png]

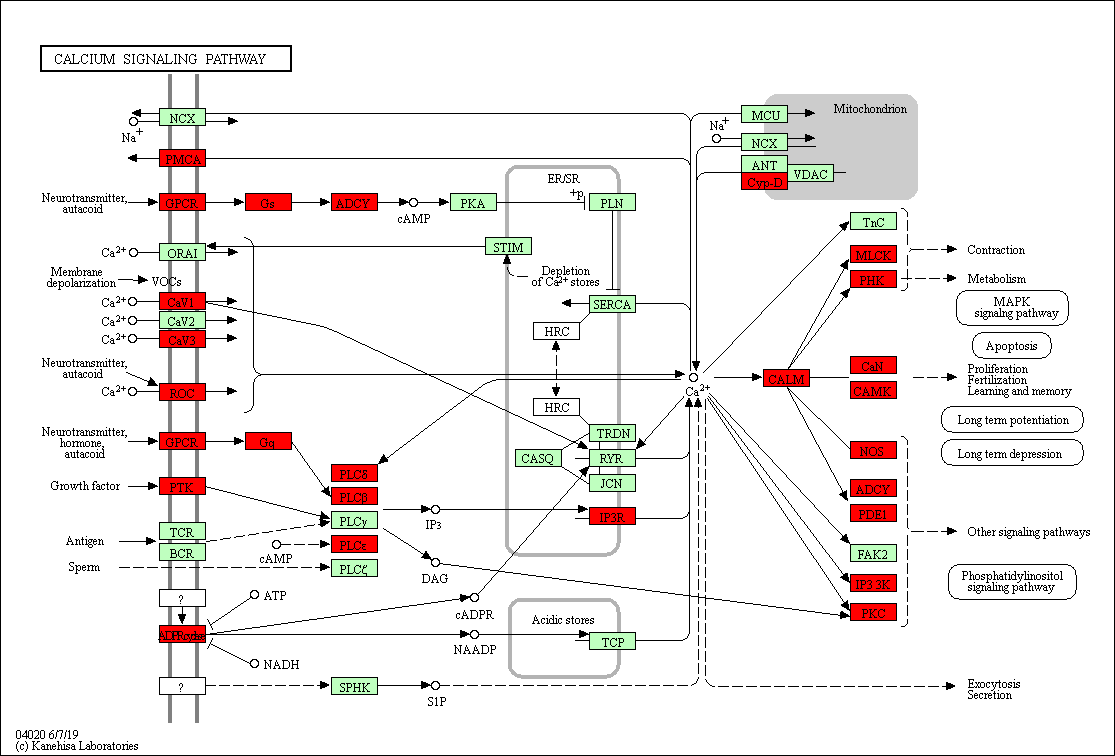

Supplement: Supplementary file 8 — Additional file 8: Fig. S3: DEGs involved in the calcium signaling pathway, which were determined based on the 04020 pathway map in the KEGG database. The red box plot shows downregulated genes, and the green box plot shows upregulated genes in the pathway (DIMFPs vs DAFPs). The figure has obtained KEGG copyright permission. [file 12864_2021_7459_MOESM8_ESM.png]

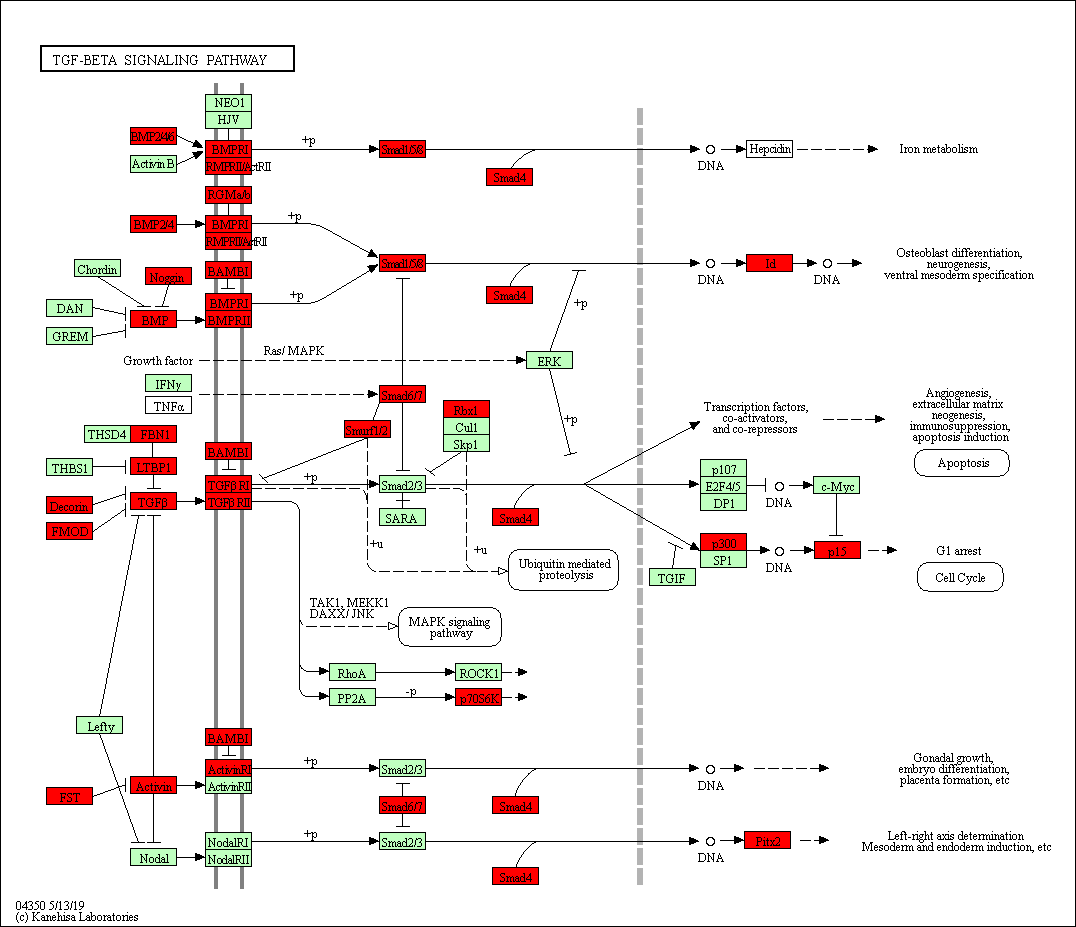

Supplement: Supplementary file 9 — Additional file 9: Fig. S4: DEGs involved in the TGF beta signaling pathway, which were determined based on the 04350 pathway map in the KEGG database. The red box plot shows downregulated genes, and the green box plot shows upregulated genes in the pathway (DIMFPs vs DAFPs). The figure has obtained KEGG copyright permission. [file 12864_2021_7459_MOESM9_ESM.png]
